# Obesity care in Chinese adults: from evidence to clinical practice

**DOI:** 10.1093/pcmedi/pbaf036

**Published:** 2025-11-28

**Authors:** Jing An, Qingyi Jia, Yan Huang, Yuzi Cao, Yaqian Duan, Huijie Zhang, Sheyu Li

**Affiliations:** Department of Endocrinology and Metabolism, Laboratory of Diabetes and Metabolism Research, West China Hospital, Sichuan University, Chengdu 610041, China; MAGIC China Centre, Cochrane China Centre, Chinese Evidence-Based Medicine Centre, West China Hospital, Sichuan University, Chengdu 610041, China; Health Promotion and Food Nutrition & Safety Key Laboratory of Sichuan Province, West China Hospital, Sichuan University, Chengdu 610041, China; Department of Endocrinology and Metabolism, Laboratory of Diabetes and Metabolism Research, West China Hospital, Sichuan University, Chengdu 610041, China; Department of Endocrinology and Metabolism, Nanfang Hospital, Southern Medical University, Guangzhou 510515, China; School of Public Health, Southern Medical University, Guangzhou 510515, China; Department of Endocrinology and Metabolism, Laboratory of Diabetes and Metabolism Research, West China Hospital, Sichuan University, Chengdu 610041, China; MAGIC China Centre, Cochrane China Centre, Chinese Evidence-Based Medicine Centre, West China Hospital, Sichuan University, Chengdu 610041, China; Health Promotion and Food Nutrition & Safety Key Laboratory of Sichuan Province, West China Hospital, Sichuan University, Chengdu 610041, China; Department of Endocrinology and Metabolism, Laboratory of Diabetes and Metabolism Research, West China Hospital, Sichuan University, Chengdu 610041, China; Department of Endocrinology, the Second Affiliated Hospital of Chongqing Medical University, Chongqing 400016, China; Department of Endocrinology and Metabolism, Zhongshan Hospital, Fudan University, Shanghai 200032, China; Department of Endocrinology and Metabolism, Laboratory of Diabetes and Metabolism Research, West China Hospital, Sichuan University, Chengdu 610041, China; MAGIC China Centre, Cochrane China Centre, Chinese Evidence-Based Medicine Centre, West China Hospital, Sichuan University, Chengdu 610041, China; Health Promotion and Food Nutrition & Safety Key Laboratory of Sichuan Province, West China Hospital, Sichuan University, Chengdu 610041, China

**Keywords:** obesity management, China, lifestyle intervention, pharmacotherapy, digital health, multidisciplinary care

## Abstract

More than 500 million Chinese adults suffered from overweight or obesity in 2023. The pandemic of obesity consumes healthcare and economic resources by imposing enormous burden from its complications such as cardiovascular, kidney and metabolic diseases. In response, China launched a series of important policy changes including “Weight Management Year”, facilitating the engagement of public health, clinical practitioners, industry and stakeholders in different fields. The shift triggered rapid evolution of technologies in obesity care including both treatment and prevention, which added great opportunities for all stakeholders. Nevertheless, challenges exist, including misdiagnosis of obesity secondary to other diseases, population disparity, indirect evidence supported by trials conducted in other ethnic groups, health inequalities and the collaboration across stakeholders with diverse backgrounds. Traditional Chinese diets such as Jiangnan Diet and activities such as Tai Chi represent tradition-based lifestyle interventions that provide Chinese people with cultural benefits. The evolution of technologies, especially digital healthcare and novel medications, will play critical roles in future obesity care in China. Policy makers and clinical and public health practitioners must make every effort to address the urgent crisis posed by obesity pandemic in China.

## Introduction

The prevalence of obesity has been increasing in China in recent decades [1]. In 2023, approximately 538 million people suffered from overweight and obesity, with an increase of 136 million from 2022 [2, 3]. The rise in obesity is particularly pronounced among rural populations, low socio-economic individuals, and younger age groups. This escalating trend poses a substantial risk of related diseases in the future decades. In response, the Chinese government has designated 2024 as the “Weight Management Year” to address this pressing health crisis.

The World Health Organization (WHO) defined obesity as a chronic disease that requires lifelong treatment [4], causing tremendous health burden in human history. In 2009, the direct medical cost for obesity accounted for over 7% of national medical costs, with additional societal losses of income from disability and reduced life expectancy [5]. By 2030, the estimated Chinese medical costs attributed to overweight and obesity are expected to account for 22% of total national medical expenses [6].

China faces major challenges in managing 484 million adults with overweight and 231 million with obesity while having limited healthcare resources compared to the western world. The country has to optimise the corporation of the health care system for the management of obesity across all levels of medical care. Clinical practice guidelines and consensuses for obesity are critical resources to keep clinicians on the same page. In 2024, the China’s National Health Commission (NHC) and the Chinese Society of Endocrinology published their first-ever clinical practice guidelines for obesity management, respectively [7, 8]. Both guidelines provide comprehensive guidance for practice, including identification, diagnosis, lifestyle modifications, medications, traditional Chinese medicine, and metabolic surgery. While Chinese clinicians have made great progress in these areas, gaps remain in localization, implementation, and surveillance of evidence-based obesity management.

## Global challenges in China for obesity care

Despite the evolution of technologies for obesity care, the world still faces challenges, including the identification and diagnosis of obesity, optimisation of treatment strategies, and long-term weight maintenance.

### Identification and diagnosis of obesity

It has been a long debate over the diagnostic tools for overweight and obesity. Although the body mass index (BMI, weight [kg]/height [m]^2^) is a widely used measure of general adiposity, it fails to distinguish fat mass from muscle mass, which may affect health in different directions. Waist circumference (WC) and waist-hip ratio (WHR) assist BMI in assessing abdominal obesity. However, the implementation of WC-based criteria is challenging due to the measurement inaccuracy in primary care. Advanced methods, including dual-energy X-ray absorptiometry (DXA), bioelectrical impedance analysis (BIA), computed tomography (CT), and magnetic resonance imaging (MRI), which provide accurate measures of fat and lean mass, are increasingly implemented in China. The discrepancy in these measure affects decision-making in people with different sexes, ages, occupations and provinces.

The diagnostic criteria for overweight and obesity in China differ from those in western countries, with cut-offs generally lower than those in the Caucasians, but similar or higher than those in the Japanese population. For Chinese adults, the most widely accepted criteria define overweight as a BMI ≥ 24 kg/m²and < 28 kg/m², and obesity as a BMI ≥ 28 kg/m². Abdominal obesity is diagnosed with a WC greater than 90 cm for men and 80 cm for women, or a WHR of ≥ 0.90 for men and ≥ 0.85 for women. For specific situations, adults with a BMI > 27.5 kg/m^2^ and obesity-related comorbidities may consider metabolic surgery, which includes most individuals classified as obese and a smaller proportion classified as overweight [7, 9]. Although some between-ethnicity studies validated some of these criteria [10], the settings remain generally arbitrary.

### Treatment optimisation to individualisation: a roadway to precision medicine

Many innovative strategies have emerged for obesity management, including dietary interventions like intermittent fasting, exercise interventions such as high-intensity interval training (HIIT), medications like semaglutide and tirzepatide, psychotherapies like cognitive behavioural therapy (CBT), and metabolic surgery. While these approaches have demonstrated efficacy at the population level, none serves as a one-pill-fits-all option, as individual responses vary significantly. The variability may serve as “effect modifiers”, which is an epidemiological term indicating factors that may influence the weight-lowering or safety effects of a treatment. This means that an individual with a given effect modifier will respond well or bad to a certain intervention. Identifying these effect modifiers poses a challenge in research, especially when multiple treatment options are available. It requests common participants, outcomes, and candidate factors. Nevertheless, the evidence remains sparse supported by the data from a very limited number of people, restricting the certainty as well as further interpretation in practice. For people with obesity, the follow-up in these studies is too short compared to the duration it takes to develop their long-term outcomes, such as cardiovascular and kidney diseases.

As a heterogeneous disease, obesity requires diagnostic criteria that encompass staging and categorisation to guide individualised treatment. Nevertheless, the current criteria fail to address staging and categorisation in practice. The NHC guideline summarises staging and categorisation systems for obesity. The categorisation strategies are based on either the pathophysiological system or clinical phenotypes [7, 11–13]. Nevertheless, none of them has yet been validated in the Chinese population to demonstrate their distribution at the population level or their capacity to guide treatment choices.

From the pathophysiological perspective, obesity can be classified into primary obesity (or simple obesity), which is largely driven by behavioural and environmental influences; and obesity secondary to other diseases, which results from identifiable endocrine, genetic, or iatrogenic causes [14, 15]. Mayo Clinic proposed a subgrouping system including abnormal satiation, abnormal satiety, emotional hunger, and hypometabolic obesity, reflecting underlying physiological and behavioural differences [12], which is friendly for the understanding of clinicians and patients. Nevertheless, the evidence for treatment responses in people among different groups remains sparse. From the phenotypic perspective, obesity with and without metabolic complications encompasses impaired insulin sensitivity and elevated cardiometabolic risk. People can be further categorised into metabolically healthy and unhealthy obesity. Sarcopenic obesity, characterised by the loss of skeletal muscle mass, can be a distinct type of obesity requiring specific rehabilitative training to improve quality of life [16–18]. Data-driven approaches facilitated by machine-learning algorithms and multi-omics integration have suggested some other obesity subgroups with distinct metabolic, genetic, and molecular signatures, thereby linking phenotype to pathophysiological pathways and clinical outcomes [13, 19, 20].

In Chinese real-world practice, the implementation of these risk stratification strategies warrants contextualisation and validation in large population-level studies. Chinese culture and social scenarios present local challenges that necessitate the localization of these category systems. Without trustworthy validation in the Chinese population for the treatment responses, attempting one treatment strategy after another may be the only pragmatic way to choose the right approach for obesity care. The coordinated care between obesity specialists and primary care doctors is a potential solution. In 2024, at least 1000 healthcare institutions launched the outpatient care specific for obesity [21], most of which adopted a multidisciplinary approach including endocrinologists, dietitians, physical therapists, psychiatrists, surgeons, and primary care doctors. Models of multidisciplinary obesity care vary widely across medical institutions, differing by team structure and care setting. Table 1 summarizes the representative roles and responsibilities of different professionals involved in multidisciplinary obesity care. Although most of these comprehensive care approaches are established in referral hospitals, some primary care centres have begun adopting them as well [22]. The increasing number of multidisciplinary cares for obesity and anecdotal evidence indicate the success, but trustworthy evidence is still lacking to confirm the effectiveness and implementation value for the practice model.

**Table 1.** Multidisciplinary model for obesity care in China.

Roles that may be included	Responsibilities
Specialist nurse/general practitioner	Health education; Documentation; Triage and referral; Screening and follow-up
Clinical nutritionist/dietitian	Nutritional guidance; Intervention assessment; Clinical nutrition therapy implementation
Endocrinologist	Endocrine-metabolic evaluation; Pharmaceutical intervention; Metabolic disorder management
Cardiovascular specialist	Cardiovascular risk assessment; Cardiovascular medication management; Cardiovascular intervention
Psychiatrist/psychologist	Mental health evaluation; Behavioral intervention; Psychological support implementation
Exercise/rehabilitation specialist	Physical activity assessment; Exercise program design; Rehabilitation intervention
Bariatric surgeon	Surgical evaluation; Metabolic surgery procedures; Perioperative care management
Traditional Chinese medicine physician	Traditional Chinese medicine syndrome differentiation; Herbal medicine prescription; Acupuncture therapy
Specialists of gastroenterology/respiratory medicine/nephrology/otolaryngology/obstetrics and gynecology/orthopedics	Assessment and management of obesity-related complications

### Stratified obesity care

Obesity consumes a high density of healthcare resources and requires significant self-care engagement. The Chinese healthcare system has to prioritise those benefits more from the obesity care, typically those with the highest burden of quality-of-life impairment and risk of death. Type 2 diabetes as a typical complication of obesity leads to further health burden including disability and death [23, 24] as well as hypertension and fatty liver [25–27]. Multiple risk staging systems facilitate the stratified care for people with obesity. The AACE staging system is one of the most widely used risk stratification systems, reflecting the impact of disease on individuals and the need for treatment [11]. The list of obesity-related complications in the staging system also facilitates the motivational interviewing and indicates the individualised treatment goal, which is understandable for patients themselves. The Lancet commission of obesity categorised people into preclinical and clinical obesity based on the presence of complications of obesity [28]. The list of complications includes both metabolic and non-metabolic diseases reflecting the burden of the disease and the motivation of the individual to change the status of the body composition. Multiple retrospective studies confirmed the discrimination of this staging system for prognosis in Chinese adults. Cardiovascular kidney and metabolic (CKM) syndrome is a risk stratification system for the general population in preventing cardiovascular diseases proposed by the American Heart Association (AHA) and the American College of Cardiology (ACC) [29]. By including risk calculators considering BMI and adiposity, CKM staging also represents a possible risk stratification system for people with obesity in China and other countries. In practice, clinicians should prioritise people with established comorbidities or those with higher risks with fatal ones for more intensive treatment such as anti-obesity medications with shared decision making [30]. Nevertheless, for older people, competing events such as malignancy should be taken into consideration for more aggressive strategies. For young adults, the subclinical irreversible lesions caused by accumulated exposure to excessive adiposity should be taken into account, such as metabolic memory [31].

## Local challenges in China for obesity care

China faces significant challenges in obesity care, along with the whole procedure of obesity care, including the recognition, differential diagnosis, and treatment engagement.

### Misdiagnosis of obesity secondary to other diseases

The differential diagnosis is essential but sometimes overlooked in clinical practice, especially for secondary pathological causes of obesity, which do not fall in the routine management of obesity. Pathological causes leading to obesity include hypothyroidism, Cushing’s syndrome, eating disorders, and many others. Standard obesity treatments are unlikely to be effective if the underlying cause is not addressed. Unfortunately, few up-to-date reviews describe how to recognise these conditions [32], which is challenging in primary care settings, especially for non-endocrine specialists. For example, the median time to initial diagnosis of Cushing syndrome is 34 months from the symptom onset [33]. Only 48% of people with hypothyroidism or subclinical hypothyroidism received appropriate thyroid hormone replacement [34]. These delays can significantly affect diagnosis and treatment of obesity-related outcomes, highlighting a critical capacity gap in identifying and managing secondary obesity causes within primary healthcare settings.

### Indirect evidence supporting obesity care in China

Although many high-quality trials on obesity treatment have been published in recent years, Chinese guidelines mainly cite Western studies. However, China’s dietary landscape is highly diverse, with eight major regional cuisines and numerous local specialties. For most Chinese people, food is not only a physiological need but also a hobby and a social behaviour, intertwined with cultural traditions, social interactions, business, and emotional expression. The “drinking culture” in business banquets, the “tradition of food persuasion” in family gatherings, and the “food customs of festivals” with local characteristics lead people with obesity to regard diet as an important social tool and a carrier of emotional expression. Adults with obesity are more likely to rely on food for leisure, self-expression, or even their work. Preferences like oily Sichuan cuisine or delicate Cantonese soups, along with wide variation between provinces and families, leads to western diets such as the Mediterranean or the Dietary Approaches to Stop Hypertension (DASH) diets are often impractical. Therefore, clinicians or dietitians need to engage patients in discussions to set realistic goals that work with, not against, ingrained food habits. Clinical implementation requires flexible strategies that combine dietary assessment tools with stage-based modification frameworks (e.g. gradual whole-grain substitution, modified hotpot options). For example, for people who are fond of late-night snacks, clinicians can guide them to gradually replace barbecued food with a light soup hot pot; for those who are highly dependent on staple foods in the north, refined wheat-based foods can be gradually replaced by miscellaneous grains, which can smooth the glycaemic response while retaining the sense of dietary satisfaction.

### Health inequity of obesity care in China

Health professionals from multiple disciplines are providing obesity care in China, including endocrinologists, dieticians, physical therapists, primary care doctors, gastrointestinal surgeons, and even nonmedical professionals in salons. Nevertheless, the healthcare quality is imbalanced across disciplines and regions. For instance, exercise prescriptions are not yet well informed by clinical practice guidelines with local adaptation among qualified practitioners. Only about 1 600 medical staff have certification for exercise prescription, with a total of 3 000 licensed practitioners. This translates to one practitioner serving over 77 000 people with obesity on average, compared with about 2 000 in the United States. Most exercise-focused specialists, such as rehabilitation physicians, therapists and sports medicine surgeons, mainly focus on patients with neuropathic or musculoskeletal disorders, aiming to maintain motor functions and quality of life. Although gym coaches sometimes fill these care gaps, some people, especially women, turn to commercial quick-fix weight loss programs, which can be associated with an elevated risk of exercise related injury. Poor coordination between exercise specialists, dieticians, and physicians further weakens the effectiveness of exercise as part of a comprehensive weight loss program, leaving many patients unsupervised and at higher risk for injury and weight regain.

### Treatment engagement in community

Weight maintenance requires long-term follow-up and close monitoring during treatment. Nevertheless, the healthcare system across levels of medical institutes is not working well for obesity care, and rising patient numbers strain the limited capacity of primary care. Without a standardised workflow, obesity remains excluded from the healthcare network. Clinicians in the secondary and tertiary hospitals have to deal with the follow-up tasks themselves, with or without occasional collaboration with the primary care doctors. This poor coordination undermines the effectiveness of specific obesity treatments and the evaluation of their implementation.

For example, the lifestyle advice given by dieticians or exercise physicists requires after-consultation monitoring at home or the workplace. Alternatively, the consultants must organise this monitoring system, with or without digital technologies, to maintain treatment effectiveness. Duplication across institutions leads to variable effectiveness and encourages unvalidated, and sometimes harmful, weight loss practices. For example, reports have described several serious fatal and nonfatal adverse events from commercial institutes using breatharians, supported only by anecdotal evidence [35].

## Opportunities for obesity care in China

**Figure 1.**
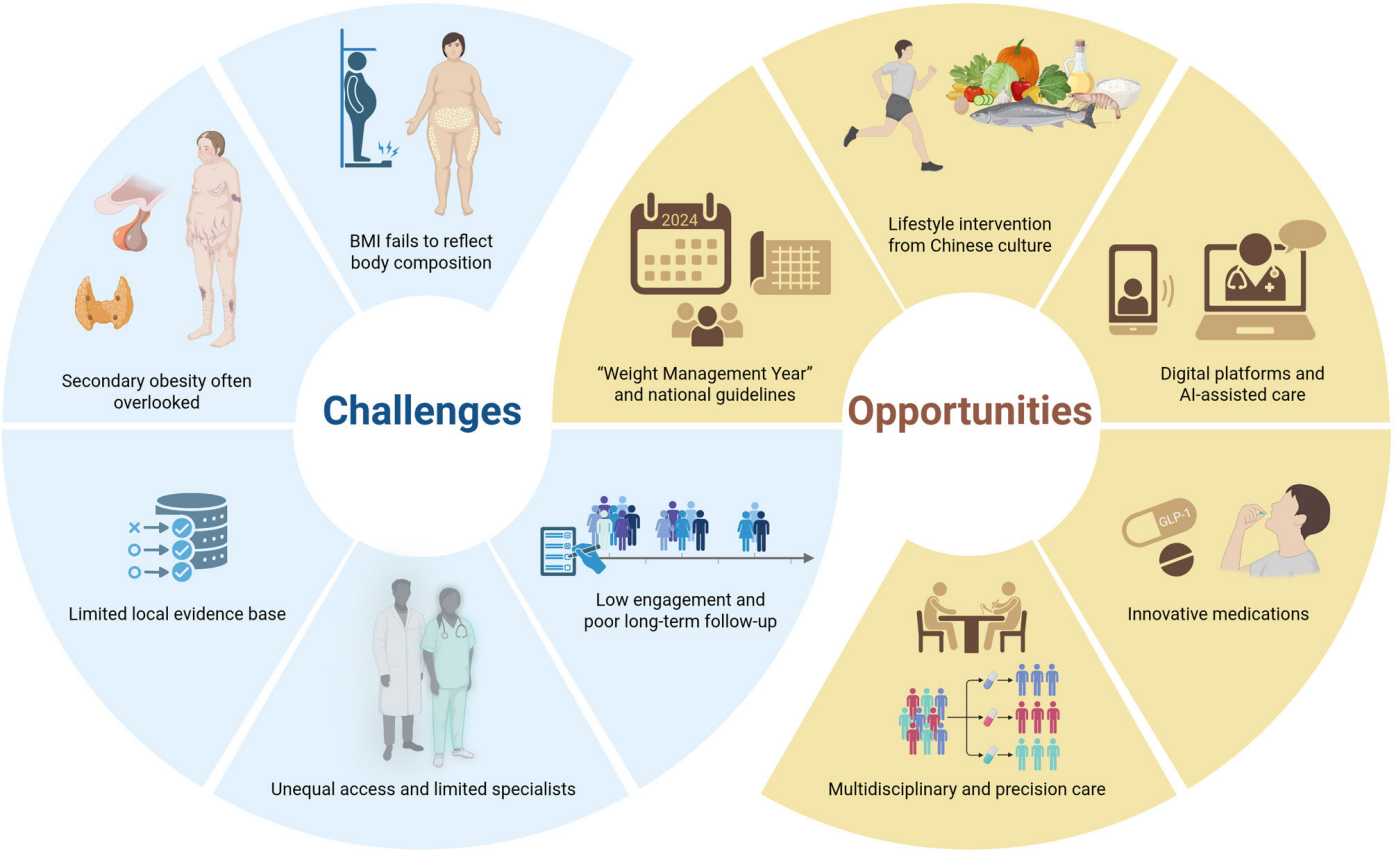
Challenges and opportunities of obesity care in China.

The challenges above also create new opportunities for innovation in obesity care (Fig. 1). Increasing awareness and policy support have encouraged culturally adapted, technology-driven, and patient-centered approaches to improve management across China.

### Chinese lifestyle for obesity care

The Chinese population has traditionally been lean. Recent studies raised several lifestyle intervention packs from the traditional Chinese culture. For instance, the traditional Jiangnan Diet, a typical traditional diet in Southeast China, represents a plant-rich diet with abundant vegetables, whole grains, soy products, and moderate freshwater fish. A six-month randomized controlled trial comparing the traditional Jiangnan Diet with the Mediterranean Diet demonstrated comparable effects on weight reduction and glucose homeostasis in adults with overweight and prediabetes [36]. Table 2 summarizes major types of dietary interventions and their clinical applications in Chinese populations. Tai Chi, a traditional Chinese form of martial art, also proved effective for body weight loss and protection against cardiovascular and metabolic diseases, as well as good tolerance and safety, especially in older people [37, 38].

**Table 2.** Summary of dietary intervention trials in China.

Type of diet	Characteristics	Target population	Clinical applications	Limitations
Calorie-controlled diet	Limits total calorie intake to 30% of energy requirements (about 600 kcal/day), mainly low-fat foods	General population	Reduces body fat percentage, improves lipid metabolism	Not suitable for individuals with growth and development needs
Very low-calorie diet	Daily intake of 600–800 kcal, aimed at rapid fat loss, preserving lean body mass and improving fat metabolism	Obese individuals	Short-term use, aims at rapid weight loss and improved fat metabolism.	Not suitable for long-term use
High-protein diet	Daily intake of protein higher than 20%, but generally no more than 35% of total energy.	Individuals needing to preserve muscle mass, such as athletes	Supports fat loss, protects lean body mass, and helps preserve muscle mass	Not recommended for those with renal issues or high protein metabolism needs
Intermittent fasting	Involves restricted eating windows (e.g. 5:2 method, eat for 5 days, fast for 2), where caloric intake on fasting days is kept low, generally around 500–600 kcal.	Overweight individuals or those with metabolic disorders	Helps control appetite, improve metabolism, and reduce fat mass	Long-term effects need further study; may be challenging for individuals with eating disorders
Low-carb diet	Focuses on a low carbohydrate intake, with carbohydrate intake generally between 20–50 g per day.	Individuals with obesity, metabolic syndrome, or diabetes	Effective for weight loss, reduces blood sugar and insulin levels, and may improve cardiovascular health	Needs careful monitoring of nutrient intake, especially fiber
Low-fat diet	Focuses on reducing fat intake, with fat intake at 20%–25% of total calories and saturated fat intake under 50g.	Individuals needing to manage obesity, hyperlipidemia, or other cardiovascular risks	Reduces total body fat, prevents further fat accumulation	May result in lower nutrient absorption, especially fat-soluble vitamins
Meal peplacement diet	Uses commercial meal replacement products (e.g. shakes, bars) to reduce overall calorie intake while maintaining balanced nutrients.	Individuals needing weight loss without hunger	Facilitates calorie control, simplifies weight loss, and ensures nutritional balance	Long-term reliance may lead to nutrient imbalance or unsustainable habits

### Digital health for obesity care

Digital health technology is becoming a crucial component of obesity care in China. At the policy level, the Healthy China 2030 Planning Outline and the Guidelines for Promoting the Development of “Internet + Healthcare” both instructed the integration of internet technologies (e.g. teleconsultation, mobile health platforms, remote monitoring) into the healthcare delivery chain via cloud platforms and referral-networks [39, 40]. The connection across multidimensional data has driven the rapid evolution of connected data systems and new healthcare modes, facilitating the medical interview, information collection, and data exchange across different stakeholders in the healthcare system, which may greatly improve the identification of diabetes, and long-term management of obesity in both community and referral hospitals.

At the clinical level, digital health tools are becoming part of routine obesity management. Tech companies have launched Internet-of-Things (IoT) devices such as smart bands and bioimpedance-based scales that monitor sleep, body composition and physical activity to generate longitudinal datasets via mobile apps. These datasets enable dietary and exercise tracking in the household setting [41]. Healthcare providers partner with digital companies to deliver remote services through platforms such as Huayitong, which based on the informatics system of West China Hospital, a prestigious and well-known medical centre located in western China, provides online consultations and regulated face-to-face interviews. Studies have shown that mobile-based weight-management programs can achieve significant weight reductions and improve glycaemic control, supporting the feasibility and affordability of digital health in diverse regions [42]. Research groups are developing systematic quality assessments of mainstream apps for obesity management to guide clinical recommendations and patient use [43].

**Figure 2.**
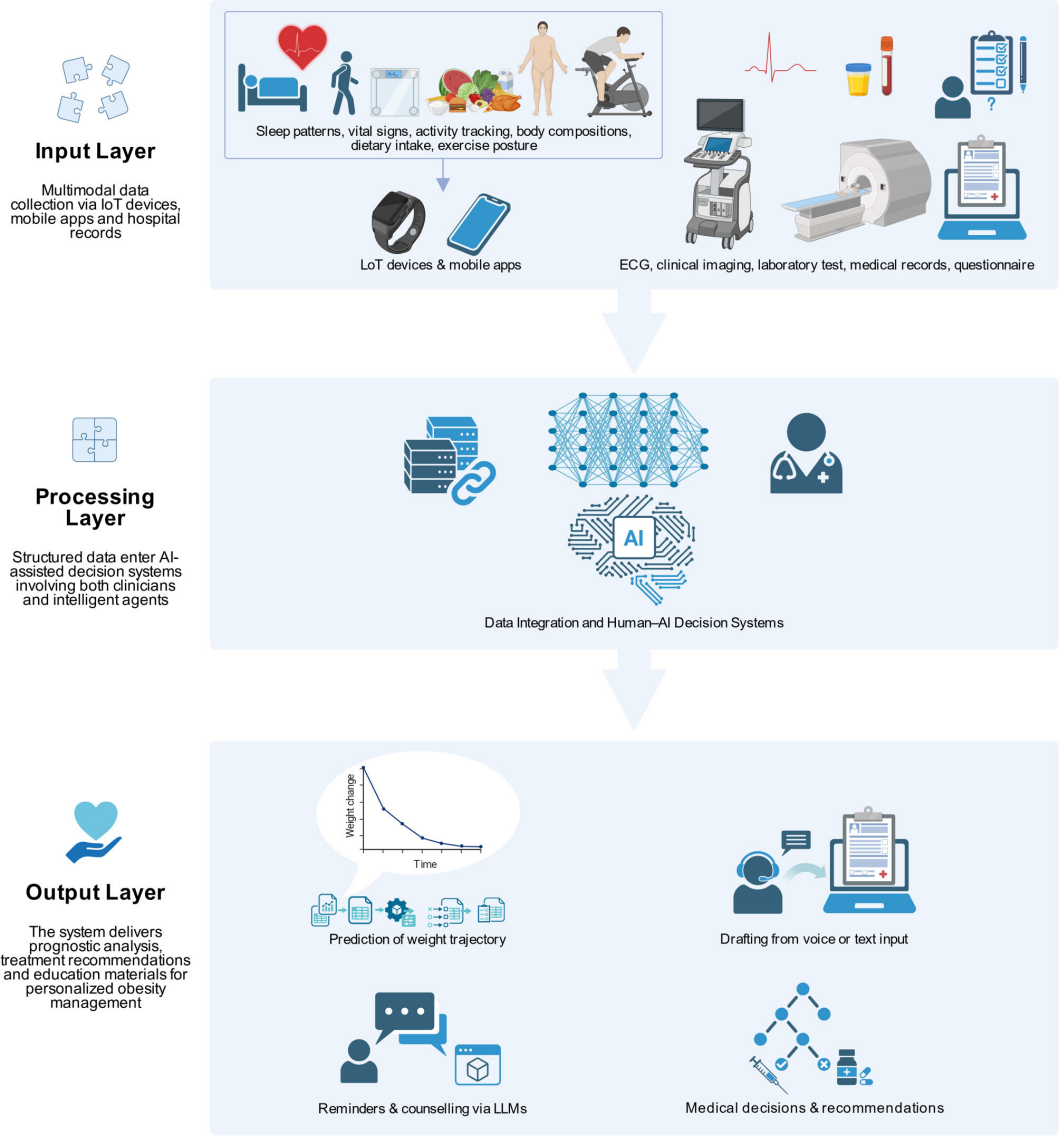
Workflow of multimodal data integration and AI-assisted decision support in obesity care.

Building on both policies and clinical practice, artificial intelligence (AI) uses vast digital datasets generated by connected health platforms to enable personalized, predictive, and adaptive obesity care in China [44]. The datasets provide the data foundation for several major technological routes of AI applications described below (Fig. 2).

With a consistently lowering technical gap for real-world practice, conversational assistants and image-recognition tools offer convenient ways to manage daily routines. Clinicians and health systems increasingly rely on AI-driven chatbots for routine guidance, appointment reminders, and medication adherence. Early systems relied on scripted dialogue trees, whereas recent large-language-model (LLM) platforms can sustain open conversations and generate context-specific counselling messages [45]. In Chinese hospitals, intelligent guidance chatbots are acceptable to both patients and clinicians and reduce waiting times [46]. LLM-based assistants are also being evaluated for psychological support and lifestyle coaching in long-term weight-management programs, aligning with evidence that conversational AI can mitigate cognitive load for clinicians and enhance continuity of care. In addition, image-recognition algorithms trained on Chinese food datasets can estimate calorie and nutrient content directly from photographs, enabling more accurate and less burdensome dietary tracking [47]. Other vision-based models reconstruct 3D body geometry to predict fat and muscle distribution [48]. Pose-estimation networks evaluate physical activity form and safety, providing real-time feedback during home exercise or rehabilitation [49]. Mobile platforms and Internet-hospital portals now incorporate these functions, giving patients practical tools for self-monitoring while generating structured data for clinicians.

Prediction models inform the baseline risks at the individual level and support clinical decision making by identifying those with urgent needs of care, while multimodal integration can reduce documentation workload. Using longitudinal anthropometric and behavioural data, deep neural networks can predict weight-loss outcomes, metabolic improvements, or the risk of weight regain. Researchers are developing similar approaches to optimize pharmacotherapy selection and to schedule follow-up intensity in outpatient obesity clinics. Compared with conventional regression methods, these models capture complex non-linear relationships between diet, activity, and physiology, facilitating the individual-level shared decision making in practice [50] and prioritising people with the highest risk and the best treatment response. Intelligent agencies assisting the preparation of clinical documents including medical records and discharge summaries improve the efficiency of clinicians’ daily practice by collecting interview records and structured information from the electronic medical records [51]. Multimodal systems combining structured laboratory data, narrative notes, and sensor streams provide decision support and anomaly alerts, and link endocrinology, nutrition, and psychology departments, reinforcing multidisciplinary obesity management. Nevertheless, the technical evolution calls for health policies including privacy protection in parallel.

Despite rapid progress, challenges remain. Data standardisation and interoperability between hospital and commercial platforms remain limited; privacy and regulatory frameworks are still evolving; and most algorithms have been trained on non-Chinese datasets, limiting external validity. Nevertheless, AI is moving from isolated demonstrations to daily clinical use. With coordinated efforts among healthcare institutions, technology companies, and regulators, these tools could underpin an evidence-based, patient-centred digital obesity-care ecosystem in China [44].

### Innovative medications

With continuously evolving biotechnology, several Chinese pharmaceutical industries have chimed in on this “Novo-Lilly campaign” with tremendous profits. Beinaglutide, developed and manufactured in China, was approved for adults with obesity in 2023. Multiple pharmaceutical companies, including local startups and those collaborating with international giants, are advancing promising drug candidates through well-designed randomised controlled trials for novel obesity medications. Mazdutide, a once-weekly glucagon-like peptide-1 (GLP-1) and glucagon receptor dual agonist, demonstrated good safety and clinically meaningful body weight reduction in Chinese adults with overweight and obesity (−11.3% compared with 1% in placebo) [52]. RAY1225, a novel once-every-two-weeks GLP-1 and GLP receptor dual agonist, showed good tolerability and induced robust body weight reduction in Chinese adults with overweight and obesity (−13.05% compared with −3.62% in placebo) [53]. The longer dosing interval may facilitate better treatment adherence.

## Summary

China, with its large population and cultural and lifestyle diversity, is facing an obesity pandemic ahead of many other countries. Chinese clinicians are now defending the complications of obesity by early identification, precision diagnosis and corresponding treatment. AI and other new technologies facilitate the evolution of obesity management in the country. Nevertheless, barriers remain including the difficulty in categorisation and surveillance.
